# Social Communication and Theory of Mind in Boys with Autism and Fragile X Syndrome

**DOI:** 10.3389/fpsyg.2012.00266

**Published:** 2012-08-20

**Authors:** Molly Losh, Gary E. Martin, Jessica Klusek, Abigail L. Hogan-Brown, John Sideris

**Affiliations:** ^1^Roxelyn and Richard Pepper Department of Communication Sciences and Disorders, Northwestern UniversityEvanston, IL, USA; ^2^Frank Porter Graham Child Development Institute, University of North Carolina at Chapel HillChapel Hill, NC, USA; ^3^Division of Speech and Hearing Sciences, Department of Allied Health Sciences, University of North Carolina at Chapel HillChapel Hill, NC, USA

**Keywords:** autism, fragile X syndrome, pragmatic language, social communication, theory of mind

## Abstract

Impairments in the social use of language, or pragmatics, constitute a core characteristic of autism. Problems with pragmatic language have also been documented in fragile X syndrome (FXS), a monogenic condition that is the most common known genetic cause of autism. Evidence suggests that social cognitive ability, or theory of mind, may also be impaired in both conditions, and in autism, may importantly relate to pragmatic language ability. Given the substantial overlap observed in autism and FXS, this study aimed to better define those social-communicative phenotypes that overlap in these two conditions by comparing pragmatic language ability and theory of mind in children with idiopathic autism and children with FXS, with and without autism, as well as children with Down syndrome and typically developing controls. We further examined correlations between these cognitive-behavioral phenotypes and molecular genetic variation related to the Fragile X Mental Retardation-1 gene (*FMR1*) in the FXS group. Results indicated that children with idiopathic autism and those with FXS and autism performed comparably on direct-assessment measures of pragmatic language and theory of mind, whereas those with FXS only did not differ from controls. Theory of mind was related to pragmatic language ability in all groups. Pragmatic language and theory of mind also correlated with genetic variation at the *FMR1* locus (Cytosine-Guanine-Guanine repeats and percent methylation). These results point toward substantial overlap in the social and language phenotypes in autism and FXS and suggest a molecular genetic basis to these phenotypic profiles.

Autism and fragile X syndrome (FXS) are genetically based neurodevelopmental disorders that share a number of cognitive and behavioral characteristics, including impairments in social communication, or pragmatic language. Pragmatic language is a complex skill grounded deeply in the capacity to apprehend and contend with social information. Mastering pragmatic language skills (e.g., politeness strategies, adopting different registers, or styles of communication depending upon addressee, conversational, and narrative practices, etc.) hinges on the ability to anticipate others’ interests, infer the background knowledge brought by each interlocutor to the communicative interaction, monitor participants’ involvement, and appreciate cultural conventions for social and communicative interaction (Grice, 1975; Brown and Levinson, 1987; Sperber and Wilson, 2002; Wilson and Sperber, 2004). Such abilities may be considered aspects of social cognition or “theory of mind,” namely, the ability to attribute thoughts, emotions, beliefs, and desires to others, and to appreciate that others may hold thoughts and feelings that are different than one’s own. Important evidence for the role of theory of mind in pragmatic language ability has come from studies of autism, where autistic groups’ theory of mind difficulties appear strongly associated with the pragmatic language impairments observed in this population (Loveland and Tunali, 1993; Tager-Flusberg and Sullivan, 1995; Surian et al., 1996; Capps et al., 1998, 2000; Tager-Flusberg, 2000; Losh and Capps, 2003). Ultimately, such findings from neurogenetic populations may provide clues to the brain and gene basis of complex human traits such as social communication and theory of mind, by providing links between gene, brain, and behavior. In other words, characterizing pragmatic language impairments in autism and related neurogenetic disorders such as FXS could help to clarify how underlying genetic variation and resultant changes in brain development might give rise to specific phenotypes such as pragmatic language or theory of mind impairment.

Whereas the genetic basis of autism is complex, with the disorder still defined behaviorally (American Psychiatric Association, 1994), FXS is a monogenic X-linked disorder that is the most common inherited cause of intellectual disability (ID) and the most common known genetic cause of autism. Because FXS is more etiologically homogeneous than idiopathic autism, careful study of autism-related phenotypes in the context of this single-gene disorder can provide an important avenue for identifying pathophysiological mechanisms underlying the symptoms of autism, and informing the genetic basis of complex human skills such as pragmatic language and theory of mind.

In this study, we compared pragmatic language ability in children with idiopathic autism and children with FXS, with and without autism, in order to better define those social-communicative phenotypes that overlap in these two conditions. We further examined theory of mind in these groups, both to characterize groups’ abilities and to determine whether there exists phenotypic overlap in this important domain, as well as to examine theory of mind as a potential underpinning factor in the pragmatic language impairments in each group. As noted, strong links have been documented between pragmatic language impairment and theory of mind in autism, but to our knowledge, these relationships have not yet been studied in FXS. Finally, we examined molecular genetic correlates of pragmatic language and theory of mind in the FXS group, with the goal of detecting gene-behavior associations that may have implications for the genetic basis of social communication and theory of mind. Below we present a brief review of FXS and rationale for comparison of pragmatic language and theory of mind in autism and FXS.

## Introduction to FXS and Its Overlap with Autism

Fragile X syndrome is the most frequent known hereditary cause of ID (Dykens et al., 2000; Hagerman and Hagerman, 2002), with the full mutation estimated to occur in approximately 1 in 2,500 to 1 in 5,000 individuals (Hagerman, 2008; Coffee et al., 2009; Fernandez-Carvajal et al., 2009). On the X chromosome, an expansion of Cytosine-Guanine-Guanine (CGG) repeats in the Fragile X Mental Retardation-1 gene (*FMR1*) results in methylation (i.e., shutting down) of the gene and reduced or absent production of the Fragile X Mental Retardation Protein (FMRP). FMRP is thought to be critical for typical brain development (Devys et al., 1993; Jin and Warren, 2003), and its deficiency in FXS is believed to underlie the physical and cognitive-behavioral characteristics of the syndrome. Males with FXS typically experience moderate or severe ID (Bennetto and Pennington, 2002; Abbeduto and Chapman, 2005) and more severe impairments than females overall, because females possess one unaffected X chromosome in addition to one affected chromosome (Hagerman and Hagerman, 2002; Loesch et al., 2003; Reiss and Dant, 2003; Bailey et al., 2008, 2009). Commonly co-occurring conditions include social anxiety (Bregman et al., 1988; Hagerman, 2002; Cordeiro et al., 2011), attentional deficits (Hooper et al., 2000; Wilding et al., 2002), and autism (Hagerman and Hagerman, 2002).

Autistic characteristics observed in individuals with FXS include stereotypic and repetitive behaviors, poor eye contact, and social avoidance (Reiss and Freund, 1992; Hagerman and Hagerman, 2002). FXS is the most common known single-gene disorder linked to autism (Hagerman and Hagerman, 2002). Results of studies using gold standard diagnostic measures indicate that 20–50% of males with FXS may also have autism and as many as three-quarters may meet ASD criteria (Rogers et al., 2001; Kaufmann et al., 2004; Philofsky et al., 2004; Clifford et al., 2007; Hall et al., 2008). In addition, approximately 2–6% of individuals with autism test positive for the fragile X mutation (Hagerman, 2006).

Language development is impaired in males with FXS beyond expectations for cognitive level, with greater deficits in language production compared with comprehension (Roberts et al., 2001, 2008; Abbeduto et al., 2007; Finestack et al., 2009). Early investigations of pragmatic language in FXS reported poor topic maintenance with inappropriate responses, rambling, automatic phrases, and perseveration or repetitive language (Hanson et al., 1986; Madison et al., 1986). However, these studies included small samples and lacked comparison groups. Compared with controls with typical development or Down syndrome (DS), males with FXS have greater difficulty maintaining topics of conversation and produce more off-topic or tangential contributions to the topic (Wolf-Schein et al., 1987; Sudhalter and Belser, 2001) as well as more perseveration (Wolf-Schein et al., 1987; Sudhalter et al., 1990; Roberts et al., 2007a). Young individuals with FXS may also be less likely than MA-matched typically developing (TD) children to report actions during story retelling (Estigarribia et al., 2011) and to request clarification or additional information in the face of unclear messages from a communication partner (Abbeduto et al., 2008). Compared with individuals with idiopathic autism, males with FXS without autism produced more turns per topic and less echolalia but more perseveration in one study (Sudhalter et al., 1990).

Autism status of participants with FXS was handled differently across the studies reviewed above – individuals with FXS and comorbid autism were either excluded (Sudhalter et al., 1990; Abbeduto et al., 2008), included as a separate group (Roberts et al., 2007a; Estigarribia et al., 2011), or autism status was not reported (Hanson et al., 1986; Madison et al., 1986; Wolf-Schein et al., 1987; Sudhalter and Belser, 2001). Several studies have directly examined the role of autism in language in FXS. On global language assessments, males with FXS and comorbid autism show more severe language deficits than males without autism (Bailey et al., 2001; Rogers et al., 2001; Philofsky et al., 2004). Findings are more mixed with respect to specific language domains, however. In several studies, groups of males with FXS did not differ by autism status in either receptive or expressive vocabulary (Price et al., 2007; Roberts et al., 2007a; Kover and Abbeduto, 2010; McDuffie et al., 2012) or syntax (Price et al., 2007, 2008; Kover and Abbeduto, 2010; McDuffie et al., 2012). However, individuals with FXS and comorbid autism performed more poorly than those with only FXS in receptive vocabulary and syntax in one study (Lewis et al., 2006), and autism severity may be negatively related to receptive vocabulary skill when a continuous analytical approach is taken (McDuffie et al., 2012). In two studies, boys with both FXS and autism did not differ from those without autism but did differ from TD controls (whereas boys with only FXS did not) in expressive vocabulary (Roberts et al., 2007b) and overall story retelling ability (Estigarribia et al., 2011), perhaps suggesting that autism in FXS negatively impacts these language areas as well. Boys with FXS and comorbid ASD have been shown to produce more off-topic or tangential language than boys with only FXS (Roberts et al., 2007a). Children and adolescents with both FXS and autism were also rated higher than those without autism in the current use of stereotyped utterances/delayed echolalia and reciprocal conversation on the Communication domain of the Autism Diagnostic Interview-Revised (ADI-R; Lord et al., 1994) in another recent study (McDuffie et al., 2010).

## Theory of Mind in FXS

For the most part, theory of mind performance in FXS appears to be on par with cognitive expectations, and children with FXS score comparably to children with DS or ID of unknown etiology (Mazzocco et al., 1994; Garner et al., 1999; Cornish et al., 2005). Children with FXS also perform similarly on false belief theory of mind tasks to younger, non-verbal mental age-matched TD children (Abbeduto et al., 2001). However, some studies have reported theory of mind deficits in FXS that cannot be explained by cognitive impairment. In a recent comparison of 30 boys with FXS to 15 boys with unspecified ID, Grant et al. (2007) found poorer overall performance on standard false belief tasks among the FXS group (Grant et al., 2007). Similar findings were reported by Garner et al. (1999), who found that a small group of eight boys with FXS performed significantly worse than a matched ID group on a deceptive box false belief task, although these findings may have been sporadic as no group differences were detected on a secondary false belief task (the Sally–Anne task), nor on a second-order false belief task (Garner et al., 1999).

A few studies have examined the impact of autism comorbidity on theory of mind abilities in FXS syndrome, and suggest that autism status may play a role in theory of mind ability in FXS. Lewis et al. (2006) compared non-verbal IQ-matched groups of children with FXS with and without comorbid autism, and found that the children with FXS who met criteria for autism showed worse performance on false belief tasks, despite similar cognitive ability (Lewis et al., 2006). The study by Grant et al. (2007) failed to detect differences in false belief performance among children with FXS with and without autism, although there was a non-significant trend toward poorer performance in the comorbid autism group (Grant et al., 2007).

## Rationale for the Present Study

In spite of considerable overlap between autism and FXS, and evidence that both disorders are characterized by difficulties in pragmatic language, and likely theory of mind as well (at least in those individuals with comorbid FXS and autism), few direct population comparisons exist to allow precise comparison of these populations and drawing ties between known underlying genetic variation and the social phenotypes of interest. Additionally, whether impairments in pragmatic language and theory of mind may be related in both populations is not known. This study addressed these questions by comparing pragmatic language ability and theory of mind in children with idiopathic autism, children with FXS with and without autism, children with DS (included as a comparison group to control for general cognitive delays), and TD children. Further, correlations with genetic variation at the *FMR1* locus were examined to inform the potential genetic underpinnings of pragmatic language and theory of mind profiles observed.

## Materials and Methods

### Participants

Study participants were 28 boys with idiopathic autism (autism only; ASD-O), 40 boys with both FXS and ASD (FXS-ASD), 21 boys with FXS only (FXS-O), 21 boys with DS, and 20 TD boys participating in a large-scale longitudinal study of speech, language, and social-behavioral profiles in children with neurodevelopmental disabilities. Boys with autism, FXS, and DS were recruited from the Research Participant Registry Core of the Carolina Institute for Developmental Disabilities (CIDD) at the University of North Carolina at Chapel Hill (UNC), genetic clinics, and parent support groups in the Southeastern, Eastern, and Midwestern U.S. TD boys were recruited through the CIDD Participant Registry Core, schools, and childcare centers in North Carolina. Study procedures were approved by the institutional review boards at UNC and Northwestern University.

Participants included only boys since females with FXS are less severely impaired than males (Hagerman and Hagerman, 2002; Loesch et al., 2002) and less likely to have autism (Clifford et al., 2007; Bailey et al., 2008). Upon enrollment, parents reported that all boys were combining three or more words. For all children, English was the primary language spoken in their homes. A composite score of Peabody Picture Vocabulary Test-Third Edition (PPVT-III; Dunn and Dunn, 1997) and Expressive Vocabulary Test (EVT; Williams, 1997) raw scores was used to match groups on receptive and expressive lexical skills to help ensure that any differences detected in social communication and theory of mind were not due to differences in structural language ability (see below for description of vocabulary measures and Table 1 for group means and standard deviations). Pairwise *t*-tests indicated no significant differences between groups (all *p* between 0.09 and 0.85, with the comparisons between DS vs. FXS-O and TD as well as between FXS-ASD vs. TD with *p* > 0.30). Age equivalent scores from both measures were included as covariates in all statistical models. All boys with FXS had a diagnosis of the full mutation. Boys were excluded for having an average hearing threshold greater than 30 dB HL in the better ear, determined from a hearing screening across 500; 1,000; 2,000; and 4,000 Hz with a MAICO MA 40 audiometer. Boys with DS and TD were screened for autism with the Social Communication Questionnaire (SCQ; Rutter et al., 2003) and also subsequently excluded for scoring as “autism” or “autism spectrum” on the Autism Diagnostic Observation Schedule (ADOS; Lord et al., 2001), described below. Table 1 provides background characteristics of participants in each group.

**Table 1:** **Group characteristics**.

	ASD-O	FXS-ASD	FXS-O	DS	TD
	*N* = 28	*N* = 40	*N* = 21	*N* = 21	*N* = 20
	
	Mean (SD)	Mean (SD)	Mean (SD)	Mean (SD)	Mean (SD)
	(Range)	(Range)	(Range)	(Range)	(Range)
Chronological age	9.21 (2.22)	10.55 (2.42)	9.61 (3.03)	10.86 (2.07)	4.84 (1.34)
	(4.16–12.74)	(6.58–15.07)	(6.06–14.98)	(6.81–14.86)	(3.23–8.78)
Non-verbal mental age^1^	5.88 (1.32)	5.02 (0.49)	5.44 (0.95)	5.33 (0.83)	5.49 (1.45)
	(3.92–10.50)	(3.50–6.00)	(4.42–8.25)	(4.33–8.25)	(3.58–9.17)
Expressive vocabulary age^2^	5.62 (1.59)	4.99 (0.99)	5.42 (1.56)	5.41 (1.30)	5.87 (2.14)
	(3.42–8.92)	(2.67–7.25)	(2.75–9.25)	(3.58–8.58)	(2.92–12.33)
Receptive vocabulary age^3^	5.76 (1.81)	5.67 (1.39)	6.36 (2.55)	5.18 (1.44)	6.12 (2.01)
	(3.08–10.00)	(2.42–8.83)	(3.42–13.83)	(2.42–7.50)	(2.17–11.58)
Mean length of utterance (morphemes)	4.18 (0.94)	3.49 (0.69)	3.98 (0.74)	3.14 (0.75)	4.87 (0.54)
	(2.22–5.49)	(2.18–4.88)	(2.27–4.74)	(1.76–4.76)	(4.12–6.05)

*^1^Leiter-R, age equivalent in years*.

*^2^Expressive Vocabulary Test (EVT), age equivalent in years*.

*^3^Peabody Picture Vocabulary Test – 3rd Edition (PPVT-III), age equivalent in years*.

### Assessments

Boys were tested in a quiet space in a school, home, or in a laboratory setting. The full assessment lasted approximately 4–6 h, with several breaks to prevent fatigue. Assessments were video-recorded with a Sony Digital8 video camera (Model DCR-TVR27) and audio-recorded with a Marantz portable solid-state recorder (PMD670).

#### Autism classification

The ADOS (Lord et al., 2001) was used to confirm autism in boys with ASD-O and to classify boys with FXS according to autism status. The ADOS consists of developmentally appropriate activities that are structured to provide a child with opportunities to show diagnostic symptoms of autism, and yields classifications of “autism,” “spectrum,” and “no autism.” Trained examiners coded administrations from video, with scoring based on the revised algorithms (Gotham et al., 2007, 2008). Coders included one research assistant who was reliable with an independent ADOS trainer, and one coder who was reliable with the aforementioned research assistant. Twenty-four boys with ASD-O were identified by the ADOS as having “autism” and three as having “spectrum.” One additional boy with ASD-O did not meet criteria for autism or spectrum on the ADOS, scoring 6 (ASD cutoff is 7). However, because his scores on the Autism Diagnostic Interview – Revised (Lord et al., 1994) all exceeded diagnostic cutoffs and medical records confirmed a clinical diagnosis by an independent diagnostician, he was not dropped from analyses. Thirty-three boys with FXS were identified by the ADOS as having “autism,” seven as having “spectrum,” and 21 as having “no autism.” Those meeting criteria for either autism or spectrum formed the group of boys with FXS-ASD.

#### Pragmatic language

The participants’ pragmatic language skills were assessed with the Pragmatic Judgment subtest of the Comprehensive Assessment of Spoken Language (CASL; Carrow-Woolfolk, 1999) and the Children’s Communication Checklist-Second Edition, U.S. Edition (Bishop, 2006). The Pragmatic Judgment subtest is a direct-assessment tool for examining general pragmatic language understanding and use. The examiner reads aloud a script representing a particular part of daily life, and children are either asked to judge the appropriateness of language used in a particular situation, or they are asked to provide a pragmatically appropriate response. Test-retest reliability coefficients for the Pragmatic Judgment subtest for the age ranges included in this study exceed 0.80, suggesting that this subtest is a reliable index of pragmatic language skill. Age equivalents were computed for the current study except in the case of a raw score of 0, which for analysis was considered missing. Four boys with ASD-O, two boys with DS, and one boy with FXS-ASD received a raw score of 0.

The CCC-2 was developed to measure social language use (although it also assesses structural language domains), and requires parents and/or teachers to rate a variety of communication difficulties or strengths according to how often the behavior in question is observed in everyday settings. For this study, teacher ratings were used. The checklist includes 70 items and yields 10 scaled scores. The scales of primary interest for pragmatic language assessment included the following: Initiation, Scripted Language, Context, Non-verbal Communication, Social Relations, and Interests. We also compared the Speech, Syntax, Semantics, and Coherence scales as variables of secondary interest. Scaled scores range from 1 to 19, with a higher value indicating better communication. The General Communication Composite (GCC) standard score was also calculated (ranging from 40 to 160) and based on the sum of 8 scaled scores (all except Social Relations and Interests).

#### Theory of mind

Theory of mind was assessed using one of two comparable batteries of tasks. The first version included the following tests: Perspective Taking, Diverse Desires, Diverse Belief, False Belief, Knowledge Access, and Explicit False Belief (Wellman and Liu, 2004; Slaughter et al., 2007). This version involved more complex, primarily verbal, presentation of the tasks. Results from initial assessments indicated that the tasks in the original battery were too difficult for some lower functioning children, and that the heavy verbal load impacted performance above and beyond children’s levels of social cognitive competence. Thus, more basic tasks assessing intentionality and understanding of desires were added to the battery (detailed below), and administration of the false belief tasks was also modified such that scenarios were enacted, rather than read as a story involving abstract characters, to decrease verbal and cognitive load (Flavell et al., 1983; Lewis and Mitchell, 1994; Repacholi and Gopnik, 1997; Matthews et al., 2003; Slaughter et al., 2007). It was not necessary to alter the Perspective Taking Task as the protocol was already interaction-based. Two, more basic, tasks were added to tap metarepresentational skills in children who were not capable of performing the original, more advanced battery – Simple Desires and Appearance-Reality – which have been used with children as young as 14 months and 3 years, respectively, and are described in the Appendix. Each participant received either the original or the modified battery of tasks, depending on when they were tested. All assessments were second-scored by a trained research assistant. See Appendix for further task description and scoring procedures.

To produce a single composite theory of mind score for all children (and ensure comparability across the initial and modified batteries), factor analysis scores were derived. First, the two batteries were tested in separate confirmatory factor analytic (CFA) models. Both factor models were estimated under weighted least squares using MPlus (Muthen and Muthen, 2006). The commonality of the Perspective Taking Task across both batteries provided an anchor that enabled us to ensure score equivalence across forms. This task was used to set the metric for the latent variable. The CFA for the older battery was run first. In the model of the newer battery, we fixed the factor loading and threshold parameters for the Perspective Taking Task to be equal to those parameters from the model of the older battery. Thus, estimates of the latent variable, theory of mind, were equivalent across both models. That is, a given respondent would be expected to receive the same score regardless of which form of the test he or she was given. Results indicated very good model fit for the one factor solution in both models (older form: RMSEA = 0.003, CFI = 1.00; newer form: RMSEA = 0.000, CFI = 1.00). Finally, we used the factor models to estimate and export theory of mind scores for each individual. To aid in interpretability, these scores were then standardized to have a mean of 10 and a standard deviation of 1.

#### Non-verbal cognitive ability

Non-verbal cognition was assessed with the Brief IQ composite of the Leiter-R (Roid and Miller, 1997), which includes Sequential Order, Figure Ground, Form Completion, and Repeated Patterns subtests. Age equivalents were calculated based on the published norms.

#### Structural language

Receptive vocabulary, expressive vocabulary, and expressive syntax were measured with the Peabody Picture Vocabulary Test-Third Edition (PPVT-III; Dunn and Dunn, 1997), EVT (Williams, 1997), and mean length of utterance (MLU; Brown, 1973), respectively. Age equivalents for the PPVT-III and EVT were calculated according to published norms. MLU in morphemes was calculated from 100 child utterances produced during the ADOS. The language samples were transcribed using Systematic Analysis of Language Transcripts (SALT) software conventions (Miller and Chapman, 2008) and using ELAN transcription software (Max Planck Institute for Psycholinguistics, 2002; Sloetjes and Wittenburg, 2008), which allowed transcribers to sync visual information from video recording with separate high-quality audio recordings. All transcribers achieved 80% reliability against two gold standard transcripts for each diagnostic group prior to transcribing samples for the present study. A random subset of the transcripts (10% or more from each group) was independently transcribed by a second research assistant, and morpheme-to-morpheme agreement between the original and reliability transcripts was 77% overall.

#### Molecular profile characterization in FXS

Measures of *FMR1*-related variation were derived from blood samples and included the number of CGG expansion repeats, percentage of gene methylation, and percentage of lymphocytes producing FMRP. The number of CGG expansion repeats was determined using PCR analysis to determine repeat size and Southern blot to confirm PCR results for expanded alleles. Phosphorimaging was performed to determine percent methylation. Blood smears were analyzed by immunocytochemistry to determine FMRP expression. The majority of blood samples (85%) were analyzed by Kimball Genetics, Inc., with remaining analyses completed by one of several other laboratories.

### Data analysis

#### Group comparisons

Between group differences in pragmatic language (indexed by the Pragmatic Judgment subtest of the CASL and select scales of the CCC-2) were examined using analysis of covariance (ANCOVA) models, with the following covariates: age equivalent scores for receptive and expressive vocabulary measured by the PPVT-III and EVT, respectively; MLU; and general cognitive ability measured by the Leiter-R. Planned *post hoc* contrasts were used to test for specific between group differences. Group differences in theory of mind were also examined with diagnosis as the primary predictor and PPVT-III, EVT, MLU, and Leiter-R included as covariates.

Given the large number of models, omnibus *F*-tests were adjusted using the Benjamini and Hochberg (1995) procedure to control for false discovery.

#### Genetic correlates of pragmatic language and theory of mind in FXS groups

Simple correlations were run with the FXS group as a whole (to increase power), between the genetic variables and measures of structural language (PPVT-III, EVT, MLU, and structural language subscales of the CCC), general cognition (Leiter-R), pragmatic language (CASL and pragmatic language subscales of the CCC-2), and theory of mind. Because the number of CGG expansion repeats and percent methylation were highly skewed, these variables were log-transformed prior to analyses.

## Results

### Group comparisons of pragmatic language

**Figure 1:**
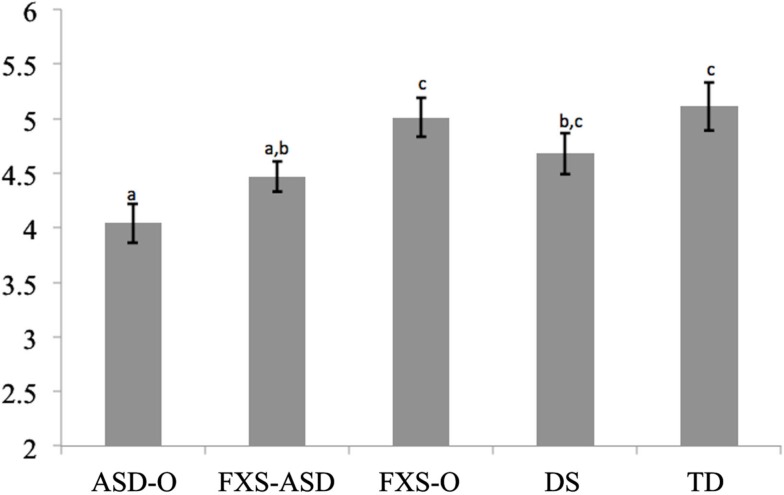
**Model Adjusted Pragmatic Judgment Scores on the CASL**. Notes: groups not sharing superscripts are significantly different from each other (*p* < 0.05). *F* for Diagnosis (4, 107) = 4.39, *p* < 0.001.

Comparisons of group performance on the Pragmatic Judgment subscale of the CASL, controlling for structural language and general cognitive abilities, were statistically significant, *F*(4, 108) = 5.49, *p* < 0.001. *Post hoc* tests (see Figure 1) indicated that the ASD-O group scored lower than the FXS-O (*d*^1^ = 0.64), DS (*d* = 0.41), and TD (*d* = 0.69) groups (*p*s < 0.05). The FXS-ASD group showed a similar pattern, with significantly lower scores than both FXS-O (*p* = 0.021, *d* = 0.35) and TD groups (*p* = 0.029, *d* = 0.41), but did not differ significantly from the DS group (*p* = 0.403). The ASD-O and FXS-ASD groups performed comparably (*p* = 0.100).

Model tests and adjusted means are presented in Table 2 for the subscales of the CCC-2. The models for the Social Relations and Interests subscales were not significant, and *post hoc* comparisons are therefore not presented for these subscales. Significant group differences were detected for all other subscales.

**Table 2:** **Model adjusted scores on pragmatic language and general language subscales of the CCC-2**.

Group	Scripted language	Initiation	Non-verbal communication	Social relations	Interests	Context	Speech	Syntax	Semantics	Coherence	CCC-2
	
											Standard Score
	
	Mean (SE)	Mean (SE)	Mean (SE)	Mean (SE)	Mean (SE)	Mean (SE)	Mean (SE)	Mean (SE)	Mean (SE)	Mean (SE)	Mean (SE)
ASD-O	5.43 (0.78)^a^	7.46 (0.76)^a,c^	4.76 (0.72)^a^	5.46 (0.71)	6.22 (0.89)	5.09 (0.65)^a^	5.97 (0.89)^a^	5.09 (0.75)^a^	6.78 (0.65)^a^	4.49 (0.77)^a^	73.56 (3.95)^a^
FXS-ASD	5.50 (0.58)^a^	4.91 (0.56)^b^	4.45 (0.54)^a^	6.31 (0.54)	6.90 (0.66)	4.80 (0.47)^a^	4.28 (0.65)^a,b^	4.33 (0.55)^a^	5.16 (0.49)^a^	3.98 (0.55)^a^	67.94 (2.85)^a^
FXS-O	4.90 (0.68)^a^	6.21 (0.63)^a,b^	4.55 (0.61)^a^	6.76 (0.59)	8.01 (0.72)	5.23 (0.54)^a^	5.77 (0.74)^a^	4.94 (0.64)^a^	5.50 (0.55)^a^	4.66 (0.62)^a^	69.64 (3.29)^a^
DS	6.70 (0.74)^a^	6.87 (0.76)^a^	6.10 (0.70)^a,b^	6.98 (0.68)	7.52 (0.85)	5.71 (0.64)^a^	3.06 (0.85)^b^	3.80 (0.72)^a^	5.73 (0.64)^a^	4.60 (0.69)^a^	72.65 (3.81)^a^
TD	9.90 (0.85)^b^	9.48 (0.83)^c^	7.86 (0.79)^b^	7.31 (0.77)	10.15 (0.97)	9.56 (0.70)^b^	9.63 (0.97)^c^	9.13 (0.81)^b^	9.07 (0.73)^b^	9.28 (0.80)^b^	96.63 (4.08)^b^
F for Diagnosis	(3, 63) = 6.36**	(3, 63) = 4.73*	(3, 64) = 4.16**	(3, 62) = 1.16	(3, 63) = 2.97	(3, 63) = 8.80**	(3, 64) = 6.45**	(3, 63) = 6.74**	(3, 64) = 4.77**	(3, 60) = 8.15**	(3, 57) = 8.58**

*Groups not sharing superscripts within a column are significantly different from each other (*p* < 0.05)*.

*p < 0.05, **p < 0.01.

The TD group scored significantly higher on the CCC-2 total score than all other groups (all *d* > 1.4) with no other between group differences. This pattern was repeated for the Syntax, Semantics, Coherence, Scripted Language, and Context subscales (all *d* for the comparison with TD > 1.3). On the Speech subscale, the DS group also had significantly lower scores than both the ASD-O (*d* = 0.34) and FXS-O (*d* = 0.76) groups, but was not different from children with FXS-ASD. TD children had higher speech scores than all other groups (all *d* for the comparison with TD > 1.3). The FXS-ASD group scored lower than both the DS (*d* = 0.65) and ASD-O (*d* = 0.71) groups on the Initiation subscale, with TD boys scoring higher than all groups but ASD-O (all *d* for the significant comparisons with TD > 0.70). The pattern of means was most notably different for the Non-verbal Communication subscale. This was the only outcome, other than Social Relations and Interests, where the DS sample did not score significantly lower than the TD sample. The TD group scored significantly higher in non-verbal communication than the ASD-O, FXS-ASD, and FXS-O groups.

### Theory of mind and pragmatic language

**Figure 2:**
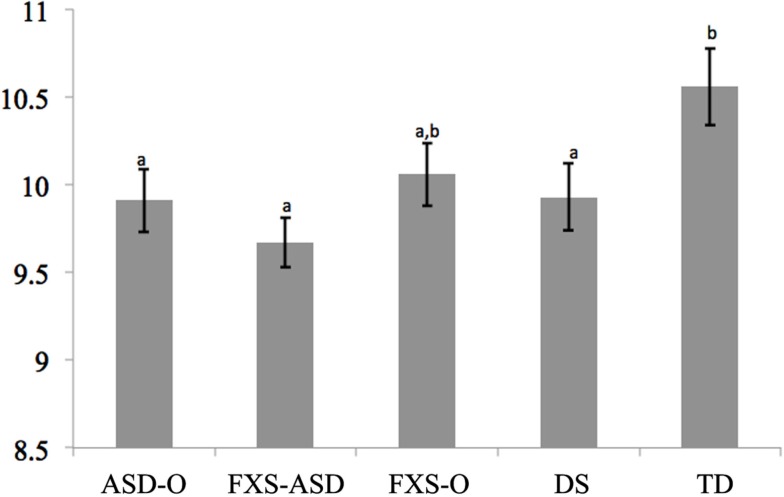
**Model Adjusted Theory of Mind Scores**. Notes: groups not sharing superscripts are significantly different from each other (*p* < 0.05). *F* for Diagnosis (4, 106) = 2.87, *p* < 0.05.

Comparing scores on the battery of theory of mind tasks, which were standardized to have a mean of 10 and a standard deviation of 1, covarying language and cognitive ability indicated that the TD group performed better than ASD-O, FXS-ASD, and DS groups (all *d* > 0.70). The difference between TD and FXS-O approached significance (*p* = 0.082, *d* = 0.56). There were no other significant group differences (see Figure 2).

Significant correlations between theory of mind and performance on the CASL Pragmatic Judgment subscale were found for all groups (see Table 3). Theory of mind was additionally related to the “Initiation” subscale of the CCC-2 in the autism group (*r* = 0.56, *p* < 0.05) and in the FXS group, it was related to the CCC-2’s “Coherence” subscale (*r* = 0.36, *p* < 0.01).

**Table 3:** **Correlations between theory of mind and pragmatic language on the CASL**.

	Theory of mind			
	ASD-O	FXS (all)	DS	TD
CASL pragmatic judgment	0.56*	0.36**	0.51*	0.54**
age equivalent (*n*)	21	57	21	19

*p < 0.05, **p < 0.01.

### Molecular genetic correlates of pragmatic language in FXS

Correlations were conducted to examine potential associations between molecular genetic variables (CGG repeat number, FMRP, and percent methylation) and measures of structural language (PPVT-III, EVT, MLU, and relevant subscales of the CCC-2), general cognition (Leiter-R), pragmatic language (Pragmatic Judgment on the CASL, and the pragmatic language subscales of the CCC-2), and theory of mind. Because CGG repeat numbers and methylation values were very skewed, these variables were log-transformed prior to analysis. Table 4 presents these results, with the exception of the CCC-2, where no significant correlations were detected. No significant associations were observed with FMRP, but higher CGG repeat numbers and increased methylation were associated with lower CASL pragmatic judgment scores. Increased methylation was also significantly related to poorer theory of mind. Measures of structural language and general cognition also showed some relationship with CGG repeat length and methylation.

**Table 4:** **Genetic correlations with language (structural and pragmatic language), general cognition, and theory of mind in the FXS group**.

	CGG Repeats	Log-transformed FMRP	Log-transformed percent methylation
PPVT	−0.33*	0.33	−0.32
*n*	36	33	33
EVT	−0.11	0.31	−0.41*
*n*	36	33	33
Leiter	−0.36*	0.34	−0.30
*n*	36	33	33
MLU	−0.35*	0.10	−0.34
*n*	36	32	32
CASL Pragmatic judgment	−0.40*	0.33	−0.36*
(*n*)	36	33	33
Theory of mind	−0.32	0.24	−0.45*
*n*	35	33	32

*p < 0.05.

## Discussion

By comparing the pragmatic language abilities of children with idiopathic autism or FXS (both with and without autism), with children with DS and TD children, this study aimed to determine the extent to which pragmatic language impairment may overlap in autism and FXS, and may potentially be tied to underlying molecular genetic variation related to *FMR1*, the gene that causes FXS. Additionally, we explored theory of mind ability as a potential correlate of pragmatic language across groups. Prior studies have reported a link between impaired theory of mind and pragmatic language use in autism, but to our knowledge this question has not yet been addressed in FXS.

Results indicated that the ASD-O and FXS-ASD groups looked quite similar on direct-assessment of pragmatic language using the CASL, with both groups performing more poorly than the FXS-O, DS, and TD groups. Yet on teacher report findings were more divergent (e.g., Initiation, where the FXS-ASD group scored significantly lower than the ASD-O group). It could be the case that a global measure of pragmatic language ability such as the CASL obscures actual differences between these groups. Alternatively, informant-based methods such as the CCC-2 may introduce measurement error that complicates group comparisons (e.g., different teachers may have different thresholds for ratings, based on their prior experience, the composition of their classrooms, etc.). Further research comparing these groups using direct-assessment measures of specific types of pragmatic language ability will be valuable in addressing this question and determining the extent of overlap in pragmatic language impairment in autism and FXS.

Analyses of theory of mind ability revealed patterns of performance quite similar to those observed in the CASL test of pragmatic language – the ASD-O and FXS-ASD groups performed most poorly, and children with FXS-O did not differ significantly from controls. In this case, however, the DS group performed more like the ASD-O and FXS-ASD groups. We also found that theory of mind ability was associated with pragmatic language on the CASL for all groups, where better theory of mind scores were associated with more pragmatic language competence. Although we cannot draw definitive causal conclusions from the present data, these findings certainly support the hypothesis that the ability to understand and predict one’s own and others’ thoughts, feelings, intentions, and desires is a critical skill underpinning competent pragmatic language use (Sperber and Wilson, 2002; Wilson and Sperber, 2004). When theory of mind is impaired, as was the case for the ASD-O and FXS-ASD groups, children may be ill equipped to contend with the demands of social discourse, and less apt to glean information necessary for developing pragmatic language skills. Such a relationship has been demonstrated across a range of pragmatic language skills in autism (Loveland and Tunali, 1993; Tager-Flusberg and Sullivan, 1995; Surian et al., 1996; Capps et al., 1998, 2000; Tager-Flusberg, 2000), and our findings suggest a similarly important role in the pragmatic language problems observed in a subgroup of children with FXS who show pragmatic language impairments as well. That significant associations were detected in all groups, even those who did not show significant pragmatic language impairment, may demonstrate the important role of theory of mind in supporting more fluent pragmatic language use as well. It is of course also possible that theory of mind tasks and pragmatic language are tapping some additional mediating (or moderating) abilities.

Patterns observed in the FXS-O and DS groups may also be informative, particularly with regard to defining syndrome-specific language and social cognitive profiles across these different groups. In particular, whereas social skills are generally considered to represent a relative strength in individuals with DS, the literature on pragmatic language in DS is actually quite mixed, with documented challenges compared with MA-matched TD children including initiation and elaboration of topics (Tannock, 1988; Roberts et al., 2007a), initiation of communicative repairs (Abbeduto et al., 2008), and clarity of messages (Abbeduto et al., 2006). Thus, our finding that boys with DS performed comparably to boys with FXS-ASD is not necessarily surprising. On the otherhand, we may have found significant differences between FXS-ASD and DS groups with a larger sample size or if we examined particular aspects of pragmatic language with direct-assessment measures (and it is important to note that the DS group did not differ significantly from the TD group, whereas the FXS-ASD group did perform significantly more poorly than the TD group). Thus, interpretation of these similarities with the present data is not straightforward.

In the FXS-O group, these data indicated that pragmatic language and theory of mind were relative strengths, and deficits in these areas may be restricted only to those with FXS-ASD, suggesting that pragmatic language deficit (or theory of mind) is not a core characteristic of FXS but rather autism in FXS. This is consistent with findings from Roberts et al. (2007a), who found that boys with FXS-O did not produce more non-contingent language than TD boys, but that the FXS-ASD group produced more non-contingent language than both of these groups. However, it is important to note that the difference between the TD and FXS-O groups approached significance so may have revealed true differences with a larger sample.

Though not a primary focus of the current study, findings do have some important clinical implications. Given that boys with FXS-ASD showed more pragmatic language impairment than boys with FXS-O, performing comparably to boys with idiopathic autism on a direct-assessment measure, the diagnosis of ASD in boys with FXS should be considered during assessment and clinicians may consider interventions that have been studied in the context of ASD when tailoring intervention approaches for boys with FXS-ASD. Our divergent findings depending on assessment method also support the use of multiple assessments, including natural language samples, to fully characterize pragmatic language ability and identify specific targets for intervention which may differ across groups and individuals.

The group similarities in directly assessed pragmatic language ability and theory of mind in ASD-O and FXS-ASD may have important implications for furthering knowledge of the brain and gene basis of these complex skills. In particular, because much is known about the molecular and neurobiological basis of FXS, the considerable overlap observed with ASD-O may help to define specific phenotypes associated with known genetic variation, in this case variation in the *FMR1*. We observed correlations with molecular genetic variables that support this association – pragmatic language on the CASL and theory of mind were both associated with *FMR1*-related variation in the FXS group. Specifically, greater methylation was associated with lower theory of mind performance and more impaired pragmatic language ability. Higher CGG repeat numbers were also related to poorer pragmatic language skills. Genetic variables showed additional associations with general cognition and structural language, which is perhaps not surprising given that general cognitive and language functioning certainly contribute to pragmatic language and theory of mind abilities. By providing a link between genetic and phenotypic variation, these findings may offer a foothold for understanding gene-behavior relationships in atypical and typical development alike.

This study has some limitations. First, we determined autism status primarily with the ADOS, but future studies should utilize information from both the ADOS and ADI-R for all participants to confirm autism status. Second, we did not examine all potential underlying mechanisms of social communication, such as anxiety or various aspects of executive function. Third, we examined social communication and theory of mind at one time point and in boys only. Future studies should assess these skills longitudinally and in both boys and girls.

In sum, this study identified pragmatic language and theory of mind as important abilities that are impaired in autism, and in a subgroup of children with FXS who also meet criteria for autism. This considerable phenotypic overlap between autism and a known monogenic condition suggests that impairments in pragmatic language ability and theory of mind may be tied to a particular genetic variant – the *FMR1*. Further studies are needed to clarify those particular types of pragmatic language difficulties common to both conditions, given that results from the pragmatic language subscales on the informant-based CCC-2 were not as straightforward as those obtained from direct-assessment of pragmatic language ability, or theory of mind for that matter. An additional important area for further study concerns the brain basis of these abilities, and the extent to which impairments may stem from similar neural architectural differences. By integrating detailed phenotypic analysis with neuroimaging studies in autism and FXS, future research may provide important insights into the role of *FMR1* in social-communicative phenotypes.

## Conflict of Interest Statement

The authors declare that the research was conducted in the absence of any commercial or financial relationships that could be construed as a potential conflict of interest.

## Footnotes

^1^Cohen’s *d* (Cohen, 1988) is a measure of effect size where 0.2 is considered small, 0.5 is considered medium, and 0.8 is considered large.

## Appendix

**Table A1:**

Task	Materials/set-up	Script	Control/test questions
Perspective taking^a^	Clear picture frame with blue fish on one side, white fish on the other. Examiner sits across from participant	“We are going to look at a picture of a fish. What color is the fish? Okay let’s switch spots. (*Examiner switches seats with participant, without moving orientation of frame*). Now what color is the fish?”	Control: none Test: “What color fish do I see over here?
Diverse desires^b^	Picture of a broccoli and cookie; female adult figurine	“Here’s Grandma. It’s snack-time. Grandma wants a snack to eat. Here are two snacks, broccoli and a cookie. Which do you like best? Well that’s a good choice, but Grandma really likes [opposite]. She doesn’t like [participant’s choice]. What she likes best is [opposite]”	Control: none Test: “Now it’s time to eat. Grandma can only choose one snack, just one. Which snack will Grandma choose?”
Diverse belief^b^	Girl figurine, displayed midway between a picture of a bush and a garage	“Here’s Amy. She wants to find her cat. Her cat might be hiding in the bushes or it might be hiding in the garage. Where do you think the cat is? Well, that’s a good idea but Amy thinks her cat is in the [opposite]”	Control: none Test: “Where will Amy look for her cat?”
False belief^b^	Goldfish crackers box with plastic toy dog inside; boy figurine	“Here’s a Goldfish box, what do you think is inside the Goldfish box? Let’s see. It’s really a dog inside! Okay, what is in the box?” “Here comes Sam. Sam has never looked inside this Goldfish box”	Control: “Did Sam look inside this box?” Test: “What does Sam think is in the box?”
Knowledge access^b^	Box with a ball inside; girl figurine	“Here’s a box. What do you think is inside the box? Let’s see… It’s really a ball inside! So, what is in the box? Here’s Amy. She’s never looked inside this box”	Control: “Did Amy look inside this box?” Test: “Does Amy know what is in the box?
Explicit false belief^b^	Picture of a backpack and closet; boy figurine	“Here’s Sam. Sam really wants to find his game. Sam’s game may be in his backpack. Or it may be in the closet. Well, really Sam’s game is in his backpack. But Sam thinks his game is in the closet”	Control: “Where is Sam’s game really?” Test: “Where will Sam look for his game?”
Unexpected contents false belief^c,d^	Cardboard M and M’s box filled with buttons. Second examiner, who has left the room	“What do you think is in this box? Lets’ look inside and see. What’s in here?”	Control: “When I first showed you the box, what did you think was inside it before you opened it?” Test: “[Second examiner] has never seen what is in this box. What will she think is in the box?”
Unexpected transfer false belief^c,d^	A pen. Second examiner places the pen on the table and announces “I need to go find my bag in the other room- I’ll leave my pen right here where it is safe”	“I know, let’s play a trick on [second examiner]. Let’s hide her pen. Where do you want to hide it?”	Control: “Where is the pen really?” Test: “When [second examiner] comes back, where will she look for her pen?”
Simple desires^e^	Bowl of Goldfish crackers and bowl of rice cakes	“It’s snack-time! Which do you like better? (*Examiner tastes each food*). Mmm [opposite of child’s preferred snack]! Mmm, I tasted the [opposite]! Mmm! Eww [child’s choice]! Eww, I tasted [child’s choice]. Eww!”	Control: none Test: “Can you give me some?” (*Examiner holds out hand*)
Appearance-reality^f^	Sponge that looks like a rock; Candle that is shaped like a crayon; Doll that is covered with a ghost cloth; White card covered by translucent pink cellophane	“When you look at this, what does it look like?	Control: none Test: “What is it really? But what does it look like?”

*^a^Slaughter et al. (2007); ^b^Wellman and Liu (2004); ^c^Matthews et al. (2003); ^d^Lewis and Mitchell (1994); ^e^Repacholi and Gopnik (1997); ^f^Flavell et al. (1983)*.
